# Reliance on condoms for contraceptive protection among HIV care and treatment clients: a mixed methods study on contraceptive choice and motivation within a generalised epidemic

**DOI:** 10.1136/sextrans-2013-051339

**Published:** 2014-04-02

**Authors:** Kathryn Church, Alison Wringe, Phelele Fakudze, Joshua Kikuvi, Zelda Nhlabatsi, Rachel Masuku, Integra Initiative, Susannah H Mayhew

**Affiliations:** 1 Department of Population Health, London School of Hygiene & Tropical Medicine, London, UK; 2 Department of Population Health, London School of Hygiene & Tropical Medicine, London, UK; 3 Family Life Association of Swaziland, Manzini, Swaziland; 4 London School of Hygiene & Tropical Medicine, Manzini, Swaziland; 5 Family Life Association of Swaziland, Manzini, Swaziland; 6 UNFPA, Mbabane, Swaziland; 7 Department of Global Health and Development, London School of Hygiene & Tropical Medicine, London, UK

**Keywords:** AFRICA, CONTRACEPTION, CONDOMS, FAMILY PLANNING, HIV CLINICAL CARE

## Abstract

**Objectives:**

To (i) describe the contraceptive practices of HIV care and treatment (HCTx) clients in Manzini, Swaziland, including their unmet needs for family planning (FP), and compare these with population-level estimates; and (ii) qualitatively explore the causal factors influencing contraceptive choice and use.

**Methods:**

Mixed quantitative and qualitative methods were used. A cross-sectional survey conducted among HCTx clients (N=611) investigated FP and condom use patterns. Using descriptive statistics, findings were compared with population-level estimates derived from Swaziland Demographic and Health Survey data, weighted for clustering. In-depth interviews were conducted with HCTx providers (n=16) and clients (n=22) and analysed thematically.

**Results:**

64% of HCTx clients reported current contraceptive use; most relied on condoms alone, few practiced dual method use. Rates of condom use for FP among female HCTx clients (77%, 95% CI 71% to 82%) were higher than population-level estimates in the study region (50% HIV-positive, 95% CI 43% to 57%; 37% HIV-negative, 95% CI 31% to 43%); rates of unmet FP needs were similar when condom use consistency was accounted for (32% HCTx, 95% CI 26% to 37%; vs 35% HIV-positive, 95% CI 28% to 43%; 29% HIV-negative, 95% CI 24% to 35%). Qualitative analysis identified motivational factors influencing FP choice: fears of reinfection; a programmatic focus on condoms for people living with HIV; changing sexual behaviours before and after antiretroviral therapy (ART) initiation; failure to disclose to partners; and contraceptive side effect fears.

**Conclusions:**

Fears of reinfection prevailed over consideration of pregnancy risk. Given current evidence on reinfection, HCTx services must move beyond a narrow focus on condom promotion, particularly for those in seroconcordant relationships, and consider diverse strategies to meet reproductive needs.

## Introduction

Literature within sub-Saharan Africa indicates that HIV diagnosis and enrolment into HIV care and treatment (HCTx) impact on contraceptive practices. While some studies find no difference in family planning (FP) use rates between HIV-positives and HIV-negatives,^1^ or no changes through seroconversion,^2^ other studies demonstrate higher rates of contraceptive use among people living with HIV (PLHIV), or increased uptake following a positive diagnosis, primarily due to greater condom use.^3–8^

Contraceptive use is also influenced by antiretroviral therapy (ART). Relatively high rates of FP use (between 46 and 85%) have been identified among those on ART,^8–11^ attributed by some to repeated contacts with health professionals.^8^ ^10^ Studies also document lower unmet FP needs among HCTx clients than testing clients,^12^ greater use of FP among those on ART than pre-ART^10^ and increases in condom use over time on ART.^13^

Factors influencing contraceptive use among PLHIV include disclosure and its effect on condom negotiation, serodiscordancy and conflicts between fertility desires and protection of negative partners, and fears about contraceptive side effects and pill burdens.^14^ ^15^ Contraceptive discontinuation and switching are also high among PLHIV using long(er) acting methods (IUD and hormonals).^16^ Studies also indicate low rates of dual method use.^5^ ^17^

Providers also play a role through negative attitudes towards child-bearing or disapproval of sexual behaviour^18–21^; concerns about health risks of pregnancy or with ART drugs^22^; fears about the impact of hormonal contraception on ART efficacy 12 15 or of IUD on infection^12^; or biases against all contraceptive methods other than condoms.^10^ ^23^

This study investigates patterns of contraceptive use among PLHIV attending HCTx services within a generalised epidemic in Swaziland, the country with the world's highest HIV prevalence (26% among adults^24^). The research formed part of a multicountry study investigating the effectiveness of service integration between sexual and reproductive health (SRH) and HIV services (the Integra Initiative^25^).

The aims of this paper are (i) to describe the contraceptive practices of HCTx clients in Swaziland, including their unmet FP needs, and compare these with population-level estimates from the Swaziland Demographic & Health Survey (SDHS); and (ii) to qualitatively explore the causal factors influencing contraceptive use among HCTx clients.

## Methods

### Quantitative HCTx survey

Methods have been described in detail elsewhere.^26^ ^27^ In summary, a cross-sectional survey of HCTx clients was conducted in 2009, among a random sample (N=611) of clients exiting HCTx services (aged ≥18, male and female, pre-ART or on ART). Sample size was determined by ability to measure a 15% difference between clinics in unmet FP needs, estimated from population-level surveys. Interviews were conducted at four HCTx clinics in Manzini, the largest town in Swaziland, ranging from fully integrated SRH-HIV to a fully stand-alone HIV clinic; these were the only clinics offering ART there at that time. The refusal rate was 15.3%.

Non-pregnant participants were asked about contraceptive use, including dual method use. To measure condom use consistency, respondents chose a statement describing their situation, including the options “I use a condom every time I have sex”, “I would like to use a condom all the time, but sometimes I don't” and “I use a condom every now and then”. Inconsistent users were defined with either of the latter two responses or by reporting no use at last sex (separate question).

Unmet FP needs were defined in three ways, among (i) married women 18–49 (the standard DHS definition of unmet need); (ii) all women 18–49 and (iii) all women 18–49, but reclassifying inconsistent condom users as having no contraceptive protection. Unmet needs were measured among pregnant women whose pregnancy was mistimed or unwanted; and among non-pregnant women not currently using a modern contraceptive method who did not want more children or none in the subsequent 2 years.

### SDHS data

The 2006/2007 SDHS individual women's and HIV data sets were merged and sample weights assigned to account for clustering. HIV status was obtained through anonymous serotesting. Unmet needs for FP were calculated according to the same definitions in the HCTx survey, except in the definition of condom consistency where available indicators differed: inconsistent users were those who reported no condom use at last sex or reported not consistently using condoms every time they had sex in the last 12 months (with their last or next to last partner). Analyses were restricted to women resident in the Manzini region (HIV-negative women N=341 and HIV-positive women N=187).

### Statistical analysis

Statistical analysis was conducted using STATA 12.0. Differences in contraceptive practices between men and women in the HCTx survey were compared using χ^2^ tests. Estimates of contraceptive use and unmet need in the HCTx and SDHS populations were compared using point estimates with their 95% CIs. Since data collection was electronic, there was only one instance of missing data from variables used in this analysis, and the population median was assigned to the case.

### Qualitative methods

Methods and an overview of client respondents have been described in detail elsewhere.^26^ ^27^ In-depth interviews were conducted with 16 providers (5 doctors and 11 nurses) and 22 clients across study clinics. Semistructured topic guides were used to explore FP practices, and provider advice and attitudes.

Qualitative sampling was both purposive and opportunistic. HCTx providers at the sites were invited to interview, with selection based on availability (all providers at three clinics, and five out of seven at the other). Clients were invited to interview during ART counselling. Efforts were made to interview both sexes, resulting in a sample of 7 men and 11 women, 5 of whom were pregnant, with a mean age of 31 (see ref. 27 for detail). Client interviews were conducted in siSwati at three time points between ART initiation and 6 months later, with six clients lost to follow-up.

Interviews were recorded and transcribed; client interviews were translated into English. Data were analysed thematically through an iterative process, including stages of (i) data familiarisation; (ii) development of coding framework (using NVivo 8.0), derived deductively from the research questions and inductively from the data; (iii) abstraction of coded data into thematic matrices; and (iv) interpretation, methodological synthesis and write-up.

## Results

### Quantitative results: patterns in contraceptive use and unmet FP needs

Table 1 shows the main characteristics of the HCTx sample. Notably, the largest groups of clients were in their 30s (38%), married or living with a partner (51%), had achieved some level of secondary education (59%), were ART refill clients (65%) and did not know partner status (39%); 18% of women were pregnant.

**Table 1:** Sociodemographic and health characteristics of study sample

Variable	Males		Females		Total		p Value (χ^2^)
	Per cent	(N)	Per cent	(N)	Per cent	(N)	
Age (group)							
<25	3.9	(5)	19.7	(95)	16.4	(100)	<0.001
25–29	7.8	(10)	27.0	(130)	22.9	(140)	
30–39	46.5	(60)	34.6	(167)	37.2	(227)	
40 or over	41.9	(54)	18.7	(90)	23.6	(144)	
Marital status							
Single	7.0	(9)	5.6	(27)	5.9	(36)	<0.001
Married	54.3	(70)	33.6	(162)	38.0	(232)	
Has partner	31.8	(41)	47.1	(227)	43.9	(268)	
Widowed/divorced/separated	7.0	(9)	13.7	(66)	12.3	(75)	
Education							
No education	11.6	(15)	6.6	(32)	7.7	(47)	0.033
0–7 years (primary)	24.0	(31)	27.2	(131)	26.5	(162)	
8–12 years (secondary)	53.5	(69)	60.6	(292)	59.1	(361)	
≥12 years (college)	10.9	(14)	5.6	(27)	6.7	(41)	
Religion							
No religion	12.4	(16)	2.1	(10)	4.3	(26)	<0.001
Protestant or Pentecostal	42.6	(55)	61.8	(298)	57.8	(353)	
Catholic	6.2	(8)	7.3	(35)	7.0	(43)	
Zionist	38.8	(50)	28.8	(139)	30.9	(189)	
Current pregnancy (or partner pregnancy)							
Yes	1.6	(2)	18.3	(88)	14.7	(90)	<0.001
Type of client							
Pre-ART	10.1	(13)	11.8	(57)	11.5	(70)	0.002
ART initiation	1.6	(2)	4.8	(23)	4.1	(25)	
ART refill	71.3	(92)	63.3	(305)	65.0	(397)	
ART user consult	17.1	(22)	11.8	(57)	12.9	(79)	
PMTCT (either pre-ART or on ART)	0.0	(0)	8.3	(40)	6.5	(40)	
Clinic attended							
Fully integrated SRH-HIV	13.2	(17)	11.4	(55)	11.8	(72)	0.009
Partially integrated SRH-HIV	15.5	(20)	30.3	(146)	27.2	(166)	
Partially stand-alone HIV clinic	36.4	(47)	28.2	(136)	30.0	(183)	
Fully stand-alone HIV clinic	34.9	(45)	30.1	(145)	31.1	(190)	
Months since clinic enrolment							
<6 months	41.1	(53)	36.9	(178)	37.8	(231)	0.245
6 months–2 years	45.7	(59)	43.6	(210)	44.0	(269)	
>2 years	13.2	(17)	19.5	(94)	18.2	(111)	
On ART							
Yes	89.1	(115)	80.1	(386)	82.0	(501)	0.017
Partner HIV concordancy							
Partner status unknown	27.9	(36)	42.1	(203)	39.1	(239)	<0.001
Partner positive	44.2	(57)	28.2	(136)	31.6	(193)	
Partner negative	16.3	(21)	8.9	(43)	10.5	(64)	
No regular partner or won't disclose their status	11.6	(15)	20.7	(100)	18.8	(115)	
Total	100.0	(129)	100.0	(482)	100.0	(611)	

ART, antiretroviral therapy; PMTCT, preventing mother-to-child transmission; SRH, sexual and reproductive health

#### Patterns of contraceptive and condom use

In total, 64% of non-pregnant respondents reported current contraceptive use (table 2). Most were modern method users (63%), but the great majority of users relied on condoms alone (80%). Men relied more often on condoms than women (87% vs 77%, p=0.03). Condoms were rarely used in addition to other methods since rates of dual method use remained very low (11%). Also, 19% of female current condom users had previously used another method, mostly injectables (79%); the two most important reasons for switching were problems with side effects (53%) and provider advice (24%) (n=38 responses).

**Table 2:** Patterns of contraceptive and condom use among females and males

	Females		Males		Total		p Value (χ^2^)
	Per cent	(N)	Per cent	(N)	Per cent	(N)	
*Among non-pregnant respondents*							
Current contraceptive use (all methods)*							
No	39.9	(157)	25.2	(32)	36.3	(189)	0.003
Yes	60.2	(237)	74.8	(95)	63.7	(332)	
Current modern method use†							
No	40.9	(161)	25.2	(32)	37.0	(193)	0.001
Yes	59.1	(233)	74.8	(95)	63.0	(328)	
Condom use vs other methods							
Condoms	76.8	(182)	87.4	(83)	79.8	(265)	0.03
Injectable	14.8	(35)	9.5	(9)	13.3	(44)	
Pills	4.6	(11)	1.1	(1)	3.6	(12)	
IUD	1.3	(3)	1.1	(1)	1.2	(4)	
Other methods	2.5	(6)	1.1	(1)	2.1	(7)	
*Non-user*		(*157*)		(*32*)		(*189*)	
Dual method use							
Single method	88.2	(209)	90.5	(86)	88.9	(295)	0.54
Dual method	11.8	(28)	9.5	(9)	11.1	(37)	
*Non-user*		(*157*)		(*32*)		(*189*)	
Total	100.0	(394)	100.0	(127)	100.0	(521)	
*Among all respondents*							
Condom use at last sex							
Condom used	69.7	(336)	80.6	(104)	72.0	(440)	0.033
Condom not used	26.6	(128)	15.5	(20)	24.2	(148)	
Not answered or missing	3.7	(18)	3.9	(5)	3.8	(23)	
Type of condom user							
Consistent user	45.0	(217)	60.5	(78)	48.3	(295)	0.029
Inconsistent user	32.8	(158)	24.8	(32)	31.1	(190)	
Never use condoms	4.8	(23)	4.7	(6)	4.7	(29)	
Not having sex	17.0	(82)	10.1	(13)	15.5	(95)	
Refused to answer	0.4	(2)	0.0	(0)	0.3	(2)	
Total	22.2	(482)	14.7	(129)	100.0	(611)	

Italicised groups are not included in p-values.

*Among men whose partner was not currently pregnant.

†Modern methods: pills, injectables, IUD, implants, female sterilisation, male and female condoms (alone), LAM.

LAM, lactational amenorrhea method; IUD, intrauterine device.

**Figure 1:**
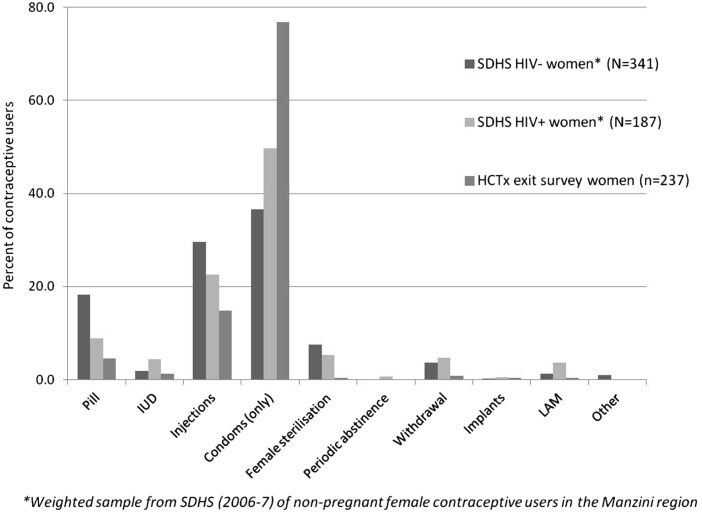
Patterns of contraceptive use among HIV care and treatment (HCTx) clients and Swaziland Demographic & Health Survey (SDHS) respondents.

Figure 1 compares contraceptive method mix among female HCTx users (N=237) with SDHS estimates among HIV-negative women (N=341) and HIV-positive women (N=187). Rates of condom use among HCTx clients (77%, 95% CI 71% to 82%) were far higher than among SDHS HIV-negative women (37%, 95% CI 31% to 43%) or HIV-positive women (50%, 95% CI 43% to 57%). Injectables were the most common method in the SDHS, used by 30% of HIV-negative and 23% of HIV-positive women, but used among only 15% of HCTx females. Pill use was also lower (18% among HIV-negative, 9% among HIV-positive SDHS and 5% among HCTx clients).

Rates of condom use at last sex were high (68% women, 81% men) (table 2). However, the combined consistent use measure demonstrated much lower rates of consistency: 45% women, 61% men.

#### Unmet FP needs

When measured among all women, including the unmarried and taking condom use consistency into account, 91 (32%) of HCTx respondents had unmet needs for FP, comprised women who were not using contraception (or inconsistently using condoms) who wanted no more children (n=46), or none within 2 years (n=17); and pregnant women whose pregnancy was mistimed (n=11) or unwanted (n=17).

**Figure 2:**
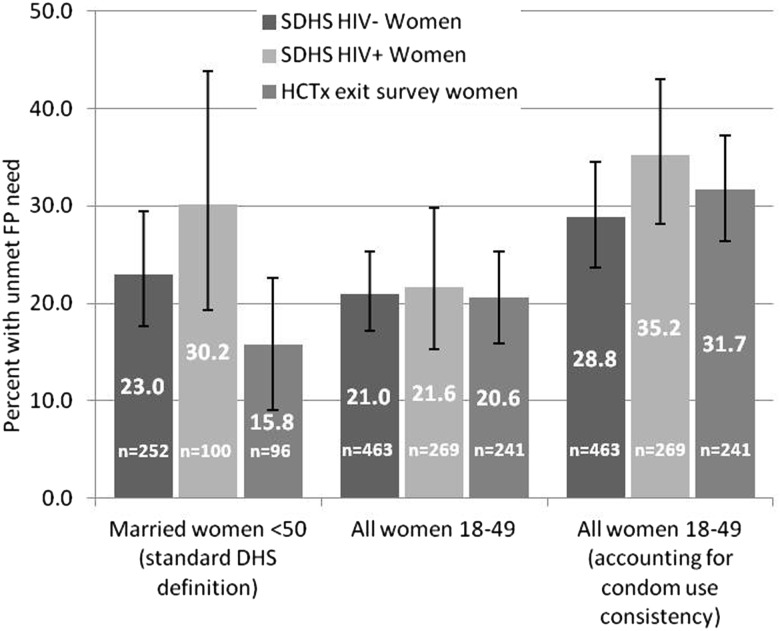
Unmet family planning (FP) needs among sexually active fecund women in HIV care and treatment (HCTx) clients and Swaziland Demographic & Health Survey (SDHS) samples.

Unmet FP needs were lower (with weak evidence) among HCTx clients compared with SDHS HIV-positive women when measured among married women (16%, 95% CI 9% to 23%; vs 30%, 95% 19–44% in HIV-positive) (figure 2). But there was no difference in unmet needs when measured among all women aged 18–49 (21%, 95% CI 16% to 25%; vs 22%, 95% CI 15% to 30% in HIV-positive) or when condom consistency was taken into account (32%, 95% CI 26% to 37%; vs 35%, 95% CI 28% to 43% in HIV-positive).

### Qualitative results: exploring causal influences on contraceptive choice

#### Condom promotion and use

High condom use rates among HCTx clients result primarily from a programmatic focus on condoms. Even at clinics where other contraceptives were available, providers considered condoms imperative to prevent viral reinfection or onwards transmission, and thus the FP method of choice for PLHIV. This messaging was clearly understood by clients, who all reiterated the providers’ advice. In some instances, counselling on condoms was the only FP advice received, despite national guidance promoting other methods. Moreover, clients often reported inaccuracies about the risks of not using condoms, derived from provider communications, including believing the virus becomes resistant or stronger, you can ‘kill’ your sexual partner, the efficacy of ARVs decreases or the risk of transmission to infants increases:

They told me that […] when I have sex without a condom the virus gets stronger […] There wasn't any [advice about FP], they just told me not to have sex without a condom because the people that I have sex with, if they don't have HIV, I'll spread it to them and also when I'm taking pills I can reinfect anybody. [Female client]

Infection concerns therefore predominated over pregnancy concerns, and other methods of contraception were considered primarily as a back-up for condoms:

they've got to use something else to prevent getting pregnant, besides the condom use, because that one is to prevent reinfection. [Provider]

Clients, in turn, expressed a notable capacity and willingness to use condoms. Motivation for condom use stemmed from a renewed personal sense of control over health after starting ART. Past inconsistent condom use was replaced by a determination to always use them:

before I started treatment, you would find that [the condoms] ran out and so we'd do it without them, but ever since I started treatment I haven't made that mistake. [Female client]

This positive response to condom promotion was even evident among those living in seroconcordant relationships. Nevertheless, concerns remained about capacity for consistent long-term condom use. Some clients did not trust condoms due to breakage concerns and many described challenges with partner compliance (mostly male, but also female). A reliance on condoms alone therefore was associated with significant concerns about the dual risks of both infecting partners and of pregnancy:

[I'm scared] about infecting [my partner…] I've tried over and over again to make him use a condom but he just won't have any of it sometimes… so now I'm just worried about getting pregnant because I don't want another baby. [Female client]

The capacity to use condoms was also strongly influenced by HIV status disclosure. Many interviewees lived in unstable informal or polygamous relationships, and were unable to disclose, encourage partner testing and/or use condoms due to abandonment fears.

#### Other contraceptives and dual method use

For some, there was little point in considering other contraceptives, and the cessation of other methods was reported due to the additional effort needed:

[my wives were] using pills but then I think they got used to using only condoms so now they're too lazy to go and get pills, or maybe they think there's no need to bother because now they know we use condoms all the time. [Male client]

Dual method use was even considered futile by some, ‘extra money and extra effort’. Others thought dual use could exacerbate men's refusal to use condoms or simply lead to increased inconsistency with condom use since they would be ‘less careful’. Cessation of other methods was also prompted by changes in sexual behaviour during ART initiation, due to illness, loss of libido and/or amenorrhoea, advice from providers, fear of infecting partners or guilt (from sexual behaviour). Consequently, clients were not always receptive to FP counselling, considered to be ‘the last of their problems most of the time’, as one provider indicated. Sexual activity, however, often resumed following ART initiation and improvement in health, and many reported getting ‘back to normal’ as CD4 levels increased. Providers noted that rapid improvements in health often left clients to be ‘caught unaware’.

Clients’ failure to use effective contraceptive methods contrasted with strong intentions to avoid pregnancy. Many were influenced by counselling from providers on the health risks of pregnancy with low CD4 counts and high viral loads, and many stressed the need for provider permission before deciding to have a child. Fears of and distress from infant HIV infection were common:

it is painful [having an HIV-infected child] because I hadn't expected him, I thought I'd stopped with the one before this one, […] which is I always get teary when I think about what will happen with the child. [Female client]

Fears of pregnancy were also compounded by fears of their own death and the implication of leaving orphans.

## Discussion

The study has demonstrated a heavy reliance on condoms for contraceptive protection among HCTx clients, greater than that documented in other studies in HCTx settings,^10^ ^11^ ^13^ including in Swaziland.^6^ When women enter HIV care, many either switch to condoms from other contraceptives or initiate condom use for infection prevention. This has resulted in a distinct contraceptive method mix to both HIV-negative and HIV-positive women in the general population. Since the SDHS sample will include many PLHIV not yet attending care, this implies that entry into HCTx results in shifts in contraceptive behaviour. Oral contraceptives and injectables, in particular, are seemingly being substituted by condoms. While further longitudinal research on contraceptive practices of PLHIV would be helpful to examine changes over time, findings here indicate that longer-term methods were considered less important, needed or appropriate for HCTx clients than condoms.

On the one hand, a high prevalence of condom use and strong client motivation to use them is an encouraging trend for positive prevention, particularly since many clients do not know partner status. Using condoms alone also carried certain benefits over dual method use, requiring less effort, while using condoms for infection control helped justify their use for pregnancy prevention. The data, however, also demonstrate that not all clients could maintain consistency, and there were consequently relatively high rates of unmet FP needs. While inclusion of data on condom consistency in the unmet need indicator is uncommon and could have resulted in elevated estimates compared with other analyses, the rates of inconsistency, and by implication unmet needs, are still likely to be underestimated since reporting bias is likely to be high in a context where respondents have been urged to use condoms.

Considering that this population maintains regular contact with health services and has strong motivations to prevent pregnancy, it is discouraging that unmet FP needs were relatively high. While the data indicate that unmet needs are lower among married women attending HCTx services, this still demonstrates a major gap in service provision given that only 38% were married. Qualitative data suggested that condom use negotiation is particularly challenging when status has not been disclosed and/or partners have not tested, both more common in unstable unions. Further attention may therefore be required to provide back-up methods for condom use failure, that is, emergency contraception or abortion (highly restricted in Swaziland).

Providers also play an important role in contraceptive choice, and data suggest they either do not understand and/or are deliberately misleading clients on the rationale for condom use. This ‘over-emphasis’ on condoms and misrepresentation of risk has been documented elsewhere in the region,^15^ and implies the need to reassess provider training on reinfection and eligibility for other FP methods. The WHO discounted the risk of ‘super-infection’ in 2008,^28^ and studies also point to the near elimination of horizontal transmission risk in those with low viral loads^29^; fears of reinfection may therefore be unwarranted. Responding to FP needs also requires adapting to clients’ changing sexual behaviour as they stabilise on treatment: a focus on FP at the time of ART initiation (also documented elsewhere^17^) may relate to a preoccupation with treatment initiation rather than long-term care needs,^30^ but is insufficient if clients are unreceptive to messaging at that time due to low libido or amenorrhoea. Greater attention to counselling is also required to address fears of contraceptive side effects, which may be particularly high in this population with other concerns about ART side effects.

The study also had further limitations. The comparison with SDHS data must be interpreted with caution since survey dates do not correspond directly (2006/7 vs 2009), although national and local service provision and policy support for FP did not change during this period. Additionally, estimates of unmet needs among PLHIV in the SDHS may be imprecise due to low sample size.

In conclusion, this study has demonstrated that a programmatic focus on condoms within HCTx settings is problematic. While it is encouraging that many clients are motivated to use condoms, capacity to sustain their use as a long-term contraceptive option is more limited. Given the distress of unwanted pregnancies among PLHIV, as well as the potential impact of FP on perinatal HIV transmission, there is thus an imperative for services to respond better to the dual risks of infection and pregnancy. Greater support must be given to HIV providers to advise on the delicate and often inseparable balance of infection and pregnancy risks faced by clients (eg, through provision of client educational materials), and back-up methods made available for those choosing condoms.

**Key messages**

- The great majority of HIV care and treatment (HCTx) clients in Swaziland rely on condoms for contraceptive protection and dual method use remains very low.
- Rates of effective contraceptive method use are lower than in general population, including among people living with HIV in the region.
- Despite regular contact with health services and strong desires to prevent childbearing, unmet family planning needs are no lower in HCTx clients than the general population.
- Fears of viral reinfection drive contraceptive choice, as well as changes in sexual behaviour before and after antiretroviral therapyinitiation.
